# Grit and Chronic Pain: Associations with Distress, Catastrophizing, Interference, and Control

**DOI:** 10.1007/s10880-025-10073-5

**Published:** 2025-03-13

**Authors:** Marc Heise, Josef I. Ruzek, Nancy Haug, Matthew J. Cordova

**Affiliations:** Palo Alto University, Palo Alto, USA

**Keywords:** Grit, Chronic pain, Resiliance

## Abstract

Individuals with chronic pain frequently experience emotional distress, negative beliefs, functional limitations, and poor sense of control. Grit, the ability to persist toward goals through passion and perseverance, has been linked to enhanced coping in chronic medical conditions but has received limited attention in the context of chronic pain. This cross-sectional study evaluated the relationship of grit to adjustment in participants with chronic pain (*N* = 58). Controlling for pain severity, conscientiousness, and neuroticism, greater grit was associated with lower pain catastrophizing (*p* = .000) and pain interference (*p* = .02) and greater life control (*p* = .02); grit was not significantly related to pain distress (*p* = .07). Prospective, longitudinal research on the relationship between grit and pain outcomes is warranted.

**Supplementary Information:**

The online version contains supplementary material available at 10.1007/s10880-025-10073-5.

## Introduction

Chronic pain is experienced by over 20% of the U.S. population (Hall, 2020; Villano et al., 2007) and is associated with emotional distress, functional impairment, and risk of disability (Pitcher et al., 2019; Treede et al., 2015). Comorbid mental health conditions (e.g., depression, anxiety, posttraumatic stress disorder) are common (Alschular & Otis, 2011; Fishbain et al., 2018; George & Beneciuk, 2015; Mallen et al., 2007; McKernan et al., 2019) and can be mutually maintaining (Nicassio & Wallston, 1992; Zautra et al., 1995). Annually in the U.S., nearly $600B is spent on chronic pain treatment (Hall, 2020; Institute of Medicine of the National Academies, 2011). Frameworks such as the biopsychosocial model (Engel, 1980; Gatchel et al., 2007) and neuromatrix theory (Melzack, 2001) suggest that psychosocial factors are prominent determinants of chronic pain’s impact.

Grit, defined as “perseverance and passion for long-term goals” despite adversity (Duckworth et al., 2007), may be related to specific indices of adjustment to chronic pain. Emotional distress is common in those with persistent pain, both due to physical discomfort and to reactions to functional limitations (Bair et al., 2003; Fonseca-Rodrigues et al., 2021; Gerrits et al., 2012). Pain catastrophizing, feeling helpless and ruminating about the threat of pain (Quartana et al., 2009), is a prominent risk factor for pain-related disability (Feldman et al., 2015; Keogh et al., 2005; Meints et al., 2017; Pitcher et al., 2019; Schütze et al., 2018). Pain interference, the extent to which pain reduces participation and satisfaction with daily activities, is an important determinant of quality of life and overall functioning (Karayannis et al., 2017) and is psychologically mediated (Doorley et al., 2021; Judge et al., 2020). Those with chronic pain can experience a reduced sense of life control as they feel unable to respond effectively to daily demands and problems due to their condition (Kerns et al., 2002). Lower life control has been linked to greater distress and disability (Oraison et al., 2023). Grit may mitigate these effects. For those with chronic pain to maintain stable mood, outlook, functioning, and sense of agency, they must persevere in the face of discomfort, set meaningful goals and work toward them in a paced way, and maintain interest in day-to-day activities despite their condition. Thus, grit may enhance coping with chronic pain, resulting in decreased distress, catastrophizing, and interference, and a greater sense of life control.

Research on grit has highlighted its salutary effects in other domains. Grit has been associated with positive outcomes in military, business, relationship, and some (Eskereis-Winkler et al., 2014) but not all (Beauvais et al., 2014; Elizonda-Omana et al., 2010; Taylor & Reyes, 2012) educational settings. Grit has been linked to greater exercise and better health (Soriano et al., 2012; Traino et al., 2019). Positive effects of grit have been found in patients with addiction (Griffin et al., 2016), Veterans with mental illness (Umucu et al., 2021), samples with various chronic medical conditions (Klappa et al., 2020, 2022; Lee et al., 2022; Moore et al., 2018; Sharkey et al., 2018; Traino et al., 2019), and mental health outcomes in the general population (Clement et al., 2020; Kim, 2015; Pennings et al., 2015).

However, the relationship between grit and responses to pain has received limited attention. In an experimental acute pain paradigm with undergraduate students (*N* = 62), grit was positively related to pain threshold and enhanced the positive relationship between resilience and time to pain threshold (Buckingham & Richardson, 2020). In a mixed sample of elite athletes and non-athletes (*N* = 71), grit was positively correlated with acute pain tolerance (Petterson et al., 2020). In pediatric and adolescent patients who had undergone orthopedic surgery for acute trauma- and sports-related injuries (*N* = 103), preoperative grit was unrelated to preoperative pain interference or intensity (Mackie et al., 2022). Among total knee and hip arthroplasty patients (*N* = 86), grit was unrelated to postoperative opioid use (Ernst et al., 2024). Similarly, grit was not related to clinical outcomes in patients who underwent spine surgery (*N* = 69; Mok et al., 2021). While grit’s impact on acute pain outcomes has been mixed, it may be most relevant to coping with chronic pain given its emphasis on persistent pursuit of goals over time. Only one published explicitly examined grit in a chronic pain sample. In that investigation, older adults with low back pain (*N* = 59) who had more grit engaged in longer duration of self-directed exercise (Kawasaki & Tozawa, 2020). Additional research on grit and chronic pain is justified and needed.

Research on grit and chronic pain should account for potential confounding factors. Grit has shown a very strong positive relationship with the personality trait of conscientiousness (Crede et al., 2017; Duckworth et al., 2007) and a moderate negative relationship with neuroticism (Duckworth et al., 2007; Lin & Chang, 2017). In turn, conscientiousness is associated with better pain outcomes (Rubén et al., 2023; Sutin et al., 2019), while neuroticism has been linked to greater pain-related distress and disability (Hanney et al., 2022; Kadimpati et al., 2015; Naylor et al., 2017). Pain severity is closely tied to affective distress and functional impairment (Rambla et al., 2023). Determining whether grit accounts for unique variance in chronic pain outcomes, above and beyond personality traits and pain severity, can inform conceptualization and care of this challenging condition.

This study evaluated the relationship between grit and coping with chronic pain in an online sample with self-reported chronic pain. It was hypothesized that, controlling for potential confounds (e.g., pain severity, conscientiousness, neuroticism), greater grit would be associated with lower pain-related distress, catastrophizing, and interference, and with greater life control.

## Method

### Procedures

Study procedures were approved by the Palo Alto University Institutional Review Board. No compensation was offered for study participation.

### Participants

Participants were recruited through postings to chronic pain groups on Reddit, Facebook, and other social media outlets. Postings invited “U.S. citizens over 18 years of age” who were “experiencing pain that has lasted longer than three months” to complete an online Qualtrics (2015) survey designed to measure “grit, resilience, and chronic pain.” To be included in the study, participants had to: (1) be 18 years of age, (2) have access to the internet, (3) be a U.S citizen, (4) read and understand English, and (5) self-report that they had experienced pain for greater than 3 months.

### Measures

#### Demographics Questionnaire

Participants provided information regarding sex, gender, age, date of birth, race/ethnicity, marital status, education, current work status, and household income. Additional questions asked participants to confirm that they had had pain for longer than three months, to report the location of their pain, and to endorse treatments received.

#### GRIT-S

The 8-item GRIT-S (Duckworth & Quinn, 2009) is a short form of the original 12-item GRIT-O scale (Duckworth & Quinn, 2009). The scale measures perseverance as an individual difference score. Questions are answered on a 1 (*very much like me*) to 5 (*not like me at all*) Likert scale and scores can range from 8 to 40; higher scores indicate greater Grit. In the current study, the Cronbach’s alpha for the GRIT-S was 0.83.

#### Pain Catastrophizing Scale (PCS)

The PCS measures catastrophizing about actual or anticipated pain, including rumination (e.g., “I can't stop thinking about how much it hurts”), magnification (e.g., “I worry that something serious may happen”), and helplessness (e.g., “There is nothing I can do to reduce the intensity of the pain”) (Sullivan et al., 1995). Thirteen items are rated on a 0 (*not at all*) to 4 (*all the time*) Likert scale. The PCS shows sound psychometrics cross-culturally (Ikemoto et al., 2019). In the current study, the Cronbach’s alpha for the PCS was 0.95.

#### West Haven-Yale Multidimensional Pain Inventory (WHYMPI)

The WHYMPI was developed to assess chronic pain from a multiple dimensional perspective (Kerns et al., 1985). It contains 52 items and 12 subscales. For this study, only section A (Pain Experience, items 1–20) was utilized. Section A yields subscale scores for: interference in areas of functioning, support from others, pain severity, life control, and affective distress. Support from others was not considered as an outcome given lack of conceptual or literature-based tie to grit. Pain severity was considered a control variable given its strong relationship to distress and disability in the literature. Participants rate the severity of their symptoms and the impact of their pain on 0 (e.g., *no interference*) to 6 (e.g., *extreme interference*) Likert scale. The WHYMPI has been used in diverse pain samples, has shown excellent test–retest reliability among people with chronic pain (Peipert et al., 2017), and is the recommended instrument for assessment of individuals experiencing chronic pain based on an evidenced-based consensus review (Dworkin et al., 2005). In this study, the Cronbach’s alphas for the WHYMPI indices included in analyses were: Interference = 0.90, Pain Severity = 0.88, Life Control = 0.72, Affective Distress = 0.83.

#### Ten-Item Personality Inventory (TIPI)

The Ten-Item Personality Inventory (TIPI; Gosling et al., 2003) is a brief measure of the Big-Five personality traits. The TIPI reached appropriate levels in terms of (a) convergence with widely used Big-Five measures in self, observer, and peer reports, (b) test–retest reliability, (c) patterns of predicted external correlates, and (d) convergence between self and observer ratings. The measure has been translated into over 30 languages and normed across male, female, and a wide-variety of racial/ethnic groups (Gosling et al., 2014). The Conscientiousness and Neuroticism subscales of the TIPI were included in the current analyses; their Cronbach’s alphas were 0.59 and 0.58, respectively.

#### Attention Check Items

Three attention check items were interspersed in the survey: “Type the day of the week” (text box); “My favorite poet is Raymond Kertezc” (true–false); “Most people would rather win than lose” (true–false).

### Data Analysis

Data were analyzed through SPSS, version 26 (IBM Corp, 2020). Data cleaning removed entries with inappropriate responses to any attention check item. Missing data were minimal (< 5%) and managed via mean imputation. Study variables were normally distributed. There was no evidence of multicollinearity in regression analyses (e.g., all VIF < 2.0). Power analysis revealed that 47 participants would be needed to have 80% probability of detecting a moderate effect size (Cohen, 1992).

## Results

A total of 86 potential participants opened the Qualtrics survey. Twenty-eight potential participants were removed due to ineligibility (*n* = 16; not a U.S. Citizen, 18 years old, or experiencing chronic pain), incomplete survey (*n* = 11), or inattentive responding (*n* = 1). Thus, the final sample consisted of 58 participants.

### Descriptive Statistics

#### Demographic and Pain Characteristics

Demographic and pain data are displayed in Table 1. Participants were in their mid-40s on average and were primarily White, cisgender, female, partnered, not working, college graduates, earning at least $50,000 annually, and non-Veterans. Pain ratings were moderate (almost 4 on a 0–6 scale). Back pain was the most frequent condition endorsed. Participants had received a variety of treatment modalities.

**Table 1:** Descriptive statistics for sociodemographic, pain, and questionnaire data (*N* = 58)

	*M*	*SD*	*n*	*%*
Age (in years)	44.9	14.2		
*Race/ethnicity*				
Black or African American			1	1.7
LatinX, Latino, or Hispanic			7	12
Asian or Asian American			2	3
Pacific Islander or Native Hawaiian			0	0
Native American, First Nation, or Alaska			2	3
White or European American			49	84.4
Arab, North African, or Middle Eastern			0	0
Not Listed			1	1.7
*Gender*				
Woman			39	67.2
Man			15	25.8
Transgender/Non-Binary			4	6.8
*Relationship status*				
Married			32	55.1
Widowed			1	1.7
Divorced			10	17.2
Separated			0	0
Never Married			15	25.8
*Work status*				
Employed full-time			14	24
Employed part-time			5	8.6
Not currently employed			3	5.1
Student			1	1.7
Retired			4	6.9
Disabled			7	12
Not Listed			1	1.7
Veteran			10	17.2
*Income*				
Less than 10,000			3	5.1
10,000–19,999			2	3
20,000–29,999			3	5.1
30,000–39,999			5	8.6
40,000–49,999			1	1.7
50,000–59,999			5	8.6
60,000–69,999			5	8.6
70,000–79,999			6	10.3
80,000–89,999			4	6.9
90,000–99,999			5	8.6
100,000–149,999			11	19
150,000-or more			8	13.8
*Education*				
Less than high school degree				
High school graduate or equivalent GED			7	12
Some college but no degree			11	19
Associates degree (2-years college)			5	8.6
Bachelor’s level (4-years college)			20	34.5
Master's Degree			14	24
Doctoral degree (Ph.D., MD, JD etc.)			1	1.7
Pain Severity	3.8	1.3		
*Treatment*				
Current medication use			47	81
Any pharmacological use			49	85
Opioid			18	31
Physical Therapy			52	90
Acupuncture			27	47
Steroid injections or Nerve blocks			44	76
Surgery			23	43
*Pain location*				
Arms, shoulder, hands			29	50
Head			26	45
Upper back			21	36
Lower back			30	52
Midsection			14	24
GRIT-S	3.49	0.73		
Pain Catastrophizing Scale	24.5	13.01		
Pain Distress	3.29	1.31		
Pain Interference	4.0	1.32		
Life Control	3.18	1.4		

Sum of percentages may be greater than 100% for variables that allowed multiple responses (e.g., race/ethnicity)

#### Primary Study Variables

Descriptive statistics for the primary study variables are shown in Table 1. Scores for pain distress, pain interference, and life control were comparable to the WHYMPI normative sample, which consisted of chronic pain patients admitted to a VA Medical Center comprehensive pain program (Kerns et al., 1985). Pain severity in the current sample was lower (~ 0.5 *SD*) than that of the WHYMPI normative sample. The mean pain catastrophizing score was lower (~ 0.5 *SD*) than a sample of Veterans in a trial of CBT for chronic pain (Murphy et al., 2022).

### Preliminary Analyses

Preliminary analyses were conducted to evaluate relationships between sociodemographic, pain, and personality characteristics and primary study variables (see Supplementary Table). Based on the pattern of preliminary results and on the literature review, pain severity, conscientiousness, and neuroticism were controlled for in hypothesis testing.

### Univariate Correlations

Univariate correlations between grit and other primary study variables are displayed in Table 2.

**Table 2:** Univariate correlations (with 95% CI) among primary study variables (*N* = 58)

	1	2	3	4	5
1. Grit	–				
2. Pain Distress	− 0.31*				
	[− 0.52, − 0.05]	−			
3. Pain Catastrophizing	− 0.40**	0.68**			
	[− 0.60, − 0.16]	[0.52, 0.80]	–		
4. Pain Interference	− 0.09	0.44**	0.64**		
	[− 0.34, 0.17]	[0.20, 0.63]	[0.45, 0.80]	−	
5. Life Control	0.38**	− 0.64**	− 0.60**	− 0.38**	
	[0.14, 0.58]	[− 0.77, − 0.46]	[− 0.74, − 0.40]	[− 0.58, − 0.14]	−

*p < .05, **p < .01

### Multiple Regression

Results of multiple regression analyses testing the four hypotheses are displayed in Table 3. Pain severity, conscientiousness, and neuroticism were entered as control variables, and grit was entered as the independent variable. Dependent variables were pain distress (Hypothesis 1), pain catastrophizing (Hypothesis 2), pain interference (Hypothesis 3), and life control (Hypothesis 4). This set of control and predictor variables accounted for significant variance in all four outcomes (all *p’s* = 0.000). Grit was not significantly related to pain distress (*p* = 0.07) but showed significant negative relationships with pain catastrophizing (*p* = 0.000) and pain interference (*p* = 0.02) and a significant positive relationship with life control (*p* = 0.02).

**Table 3:** Multiple regression analysis of grit’s effect on pain distress, catastrophizing, interference, and control, controlling for pain severity, neuroticism, and conscientiousness (*N* = 58)

Dependent	Predictors			
Variable	Grit	Conscientiousness	Neuroticism	Pain Severity
Distress				
*B*	−0.447	−0.038	0.122	0.600
*SE*	0.244	0.091	0.100	0.104
*ß*	−0.25	−0.05	0.15	0.59
*t*	−1.83	−0.41	1.21	5.79
*p*	0.07	0.68	0.23	0.000
*R*^*2*^ = 0.501, *F*(4,53) = 13.3,* p* = .000				

Catastrophizing				
*B*	−9.153	0.593	−0.213	7.09
*SE*	2.086	0.781	0.860	0.888
*ß*	−0.53	0.07	0.03	0.70
*t*	−4.38	0.76	−0.25	7.99
*p*	0.000	0.45	0.81	0.000
*R*^*2*^ = 0.632, *F*(4,53) = 22.7, *p* = .000				

Interference				
*B*	−0.556	0.145	−0.089	0.808
*SE*	0.225	0.084	0.093	0.096
*ß*	−0.31	0.17	−0.11	0.79
*t*	−2.48	1.72	−0.97	8.46
*p*	0.02	0.09	0.34	0.000
*R*^*2*^ = 0.584, *F*(4,53) = 18.6, *p* = .000				

Pain control				
*B*	0.695	0.078	−0.025	−0.492
*SE*	0.293	0.11	0.121	0.125
*ß*	0.36	0.09	−0.03	−0.45
*t*	2.37	0.7109	−0.21	−3.95
*p*	0.02	0.48	0.84	0.000
*R*^*2*^ = 0.370, *F*(4,53) = 7.8, *p* = .000				

## Discussion

Chronic pain is common, costly, associated with a range of negative psychosocial effects, and best understood within a biopsychosocial model. Grit, the ability to persist toward goals through passion and perseverance, would seem to be a resource for coping with chronic pain, but research in this realm is sparse. This study evaluated the relationship of grit to adjustment outcomes in an online sample of participants that self-identified as having chronic pain. Consistent with hypotheses, when controlling for pain severity, conscientiousness, and neuroticism, greater grit was associated with lower pain catastrophizing, lower pain interference, and greater life control. The relationship between grit and pain distress did not reach significance. Study findings, limitations, and implications for future research are discussed.

Grit did not account for unique variance in pain distress, above and beyond pain severity and personality traits (*p* = .07). Grit has been shown to facilitate efficacy in working toward long-term goals under challenging circumstances (Duckworth et al., 2007) and has been associated with better emotional adjustment to other chronic health conditions (Klappa et al., 2020, 2022; Lee et al., 2022; Sharkey et al., 2018; Traino et al., 2019). It was hypothesized that those with greater grit would experience chronic pain as less distressing because they are able to stay focused on their valued, superordinate goals in the face of this difficult condition. Given the relatively small sample size, this study may have been under-powered to detect this effect. It is also possible that the strong link between pain severity and affective distress (Rambla et al., 2023) rendered grit less impactful in multivariate analyses.

Grit was negatively related to pain catastrophizing. Catastrophizing, the tendency to ruminate, magnify, and feel helpless about chronic pain (Sullivan et al., 1995), inhibits active coping with chronic pain and has been associated with greater disability and distress (Leung, 2012). Catastrophizing is associated with negative predictions about the future, such as not being able to cope or function with pain (Quartana et al., 2009). Given that grit is associated with pursuit of long-term goals (Duckworth et al., 2007), it may help those with chronic pain shift focus away from pain and toward valued objectives and feel more empowered and efficacious. Indeed, grit has shown a positive relationship to hope and ambition in general and health samples (Clement et al., 2020; Kim, 2015; Moore et al., 2018; Pennings et al., 2015).

Grit emerged as a significant negative predictor of pain interference. Pain interference reflects the extent to which pain is an obstacle in daily functioning, including work, domestic chores, social relationships, and leisure/recreational activities (Kerns et al., 1985). Findings reinforce the possibility that grit enables those with chronic pain to persist in daily activities despite their condition (Kawasaki & Tozawa, 2020). Grit has been found to foster better executive functioning (e.g., planning, self-control, problem-solving; Liao & Chen, 2022), ability to meet challenges in educational, work, and military settings (Eskereis-Winkler et al., 2014; Sharkey et al., 2018), and achievement (Alhadabi & Karpinski, 2020; Arslan et al., 2013; Duckworth et al., 2007). Grit may help an individual focus less on pain-related negative emotions and thoughts, reduce avoidance (e.g., of movement, of difficult tasks), and more consistently pursue valued activities and goals.

Grit was positively related to life control. Life control reflects one’s perceived ability to manage life and life’s problems (Kerns et al., 1985). Poor life control is common in those with chronic pain and is associated with greater disability (Yang et al., 2019). Grit has been positively associated with coping self-efficacy (Fabelico & Bonimar, 2020) and may give those with chronic pain greater confidence in their ability to remain active and respond to problems and challenges that arise due to pain and more generally (Rusadi et al., 2021).

Whether grit is a unique construct has been questioned (Tynan, 2021). Given their strong positive correlation (Crede et al., 2017; Rimfeld et al., 2016), some have suggested that grit is a subconstruct of conscientiousness (Roberts et al., 2005; Schmidt et al., 2018, 2020). Both involve diligent pursuit of goals, although grit is distinct in its emphasis on passion about and personal meaningfulness of goals despite adversity (Ponnock et al., 2020). In this study, grit was positively correlated with conscientiousness (*r* = 0.46, *p* = .000) but accounted for unique variance in pain-related outcomes. Grit is also similar to resilience, which has been shown to bolster coping with chronic pain (Anrewasiporn et al., 2018; Fitzpatrick, 2009; Karoly & Ruehlman, 2006; Peters, 2011; Slepian et al., 2019; Sturgeon & Zautra, 2010; Yeung et al., 2012; Zautra, 2009). While resilience shares grit’s active, positive response to adversity (Tugade & Fredrickson, 2004), it does not include passionate pursuit of goals (Stoffel & Cain, 2018). The concept of committed action (Hayes et al., 2012; McCracken, 2023), dedication to living consistent with one’s values, shares grit’s emphasis on meaning but does not necessarily focus on perseverance toward goals despite obstacles. Other grit-adjacent, pain-relevant constructs include self-efficacy (Edwards et al., 2016; Kalapurakkel et al., 2015; Keefe & France, 1999; Lackner & Carosella, 1999; Lee et al., 2015; Miro et al., 2011; Rudy et al., 2003), optimism (Basten-Günther et al., 2018; Ramirez-Maestra et al., 2012; Sturgeon & Zautra, 2010), ambition, diligence, persistence, self-discipline, hardiness, dutifulness (Crede et al., 2017; Datu, 2020; Dumfart & Neubauer, 2016; Maddi et al., 2013), and self-control (Duckworth & Gross, 2014). Additional trait measures were not included in the current unfunded study given efforts to minimize participant burden; future research is needed to determine the differential effect of grit and these factors in the context of chronic pain.

This study has notable limitations. The small, demographically homogenous (e.g., mostly White, female, college-educated), social-media recruited, non-clinical sample limits generalizability. This sample may not be representative of the general population of adults with chronic pain; individuals who join health-related groups on social media may differ significantly from those who do not (Alhusseini et al., 2021). This sample exhibited similar levels of pain interference, pain distress, and life control, but lower pain severity and pain catastrophizing, compared to clinical chronic pain samples (Kerns et al., 1985; Murphy et al., 2022). Self-report measurement, particularly for a socially desirable construct such as grit, is subject to self-presentation bias; it has been suggested that grit needs to be measured by actual performance (Willingham, 2016). The cross-sectional design limits conclusions regarding causality; while grit may lead to better functioning and coping with pain, those functioning better may have simply endorsed a greater ability to persist toward goals (i.e., grit). As noted above, other than two-item indices of conscientiousness and neuroticism (both of which showed low internal consistency), measures of potentially overlapping constructs with grit were not included in this study, limiting conclusions about grit’s novelty as a coping resource for chronic pain.

Together, study findings and limitations suggest directions for future research. More research is needed to determine the extent to which grit has a unique impact on responses to chronic pain, above and beyond related constructs (e.g., conscientiousness, resilience). Crede et al. (2017) argued that grit combines passion and perseverance, and that the relative importance of each to performance and coping outcomes is unclear. Studies that evaluate these components of grit separately would enhance understanding of this construct and its import in chronic pain. Further, the grit construct may be situation- or goal-specific, is yet to be adequately behaviorally operationalized (i.e., what do people high in grit actually say, think, and do differently than those low in grit?), and may be confounded with ability to set goals. Future studies should seek larger, more diverse, clinical chronic pain samples, use prospective, longitudinal designs, assess grit regarding specific situations and goals, and employ both self-report and performance-based measures of grit.

The current study analyzed grit’s effect on pain distress, pain catastrophizing, pain interference, and life control in individuals with self-reported chronic pain. Greater grit was associated with lower catastrophizing and interference, and with greater control, even when controlling for pain severity, conscientiousness, and neuroticism. Findings highlight the importance of assessing grit in those with chronic pain and suggest directions for future investigation.

## Supplementary Information

Below is the link to the electronic supplementary material.

Supplementary file1 (DOCX 18 KB)

## Data Availability

The data that support the findings of this study are available from the corresponding author, MH, upon reasonable request.
