# Supplementary material for: Grit and Chronic Pain: Associations with Distress, Catastrophizing, Interference, and Control
Source: J Clin Psychol Med Settings. 2025 Mar 13;32(3):487–97. doi: 10.1007/s10880-025-10073-5 (PMC12370790; doi:10.1007/s10880-025-10073-5)
Supplement: Supplementary file 1 — Supplementary file1 (DOCX 18 KB) [file 10880_2025_10073_MOESM1_ESM.docx]

Supplementary Table

Correlations Among Sociodemographic, Pain, and Personality Variables and Primary Study Variables

|  | Grit | | Pain  Distress | Catastrophizing | Pain Interference | Pain Control | |  |
| --- | --- | --- | --- | --- | --- | --- | --- | --- |
| Age |  | .41^***^ | -.23 | -.13 | .01 | | .24 | |
| Gender |  | -.20 | .21 | .11 | .07 | | -.19 | |
| Race/Ethnicity |  | .17 | -.05 | -.13 | -.05 | | -.01 | |
| Household Income |  | .18 | -.02 | -.06 | -.12 | | .29^*^ | |
| Education |  | .39^*^ | -.18 | -.23 | -.08 | | .08 | |
| Employment |  | -.17 | .07 | -.001 | -.22 | | -.07 | |
| Veteran Status |  | .13 | -.29^*^ | -.33^*^ | -.33^*^ | | .33^*^ | |
| Pain Medication |  | -.09 | .30^*^ | .37^**^ | .51^***^ | | -.24 | |
| Opioid Medication |  | .06 | -.08 | .03 | .36^*^ | | .07 | |
| Pain Severity |  | .09 | .60^***^ | .64^***^ | .73^***^ | | -.43^***^ | |
| Conscientiousness |  | .46^***^ | -.25 | -.22 | -.01 | | .30^*^ | |
| Neuroticism |  | -.60^***^ | .40^**^ | .37^**^ | .15 | | -.34^**^ | |

Note. ^*^*p* < .05, ^**^*p* < .01, ^***^*p* < .001. Age (*n* = 58); Gender (*n* = 52, coded: 0 = male, 1 = female); Race/Ethnicity (*n* = 58, coded: 0 = White, 1 = Person of Color); Household Income (*n* = 58, coded: 1 = less than $10K, 2 = $10K-19999, 3 = $20K-29999, 4 = $30K-39999, 5 = $40K-49999, 6 = $50K-59999, 7 = $60K-69999, 8 = $70K=79999, 9 = $80K-89999, 10 = $90K-99999, 11 = $100K-150000, 12 = greater than $150K); Education (*n* = 38; 1 = less than high school degree, 2 = high school graduate, 3 = some college, 4 = two-year college degree, 5 = four-year college degree, 6 = masters degree, 7 = doctoral degree); Employment (*n* = 58; coded: 0 = no, 1 = yes); Veteran Status (*n* = 58; coded: 0 = no, 1 = yes); Pain Medication (*n* = 58; coded: 0 = no, 1 = yes); Opioid Medication (*n* = 48; coded: 0 = no, 1 = yes); Pain Severity (*n* = 58, rated 0-6); Conscientiousness (*n* = 58); Neuroticism (*n* = 58).
